# Novel sequential treatment with palbociclib enhances the effect of cisplatin in RB-proficient triple-negative breast cancer

**DOI:** 10.1186/s12935-020-01597-x

**Published:** 2020-10-12

**Authors:** Yajing Huang, Hao Wu, Xingrui Li

**Affiliations:** 1 Department of Breast and Thyroid Surgery, Tongji Hospital, Tongji Medical College, Huazhong University of Science and Technology, Wuhan, Hubei China; 2 Department of General Surgery, Tongji Hospital, Tongji Medical College, Huazhong University of Science and Technology, Wuhan, Hubei China

**Keywords:** Palbociclib, Cisplatin, RB, Cell cycle, Triple-negative breast cancer

## Abstract

**Background:**

Triple-negative breast cancer (TNBC) is a highly aggressive malignancy that lacks sensitivity to chemotherapy, endocrine therapy or targeted therapy. CDK4/6 inhibitors, combined with endocrine therapy, have been shown to be effective in postmenopausal women with HR-positive, HER2-negative advanced or metastatic breast cancer. Therefore, we investigated whether the CDK4/6 inhibitor palbociclib (PD) could enhance the effects of cisplatin (CDDP) on TNBC.

**Methods:**

The effects of different drug regimens consisting of PD and CDDP on MDA-MB-231 and RB-knockdown MDA-MB-231 (sh-MDA-MB-231) cells were assessed in vitro and in vivo. MDA-MB-468 and RB-overexpressing MDA-MB-468 cells were used to assess the effect of the PD-CDDP regimens in vitro. Immunoblotting illustrated the role of the cyclin D1/RB/E2F axis signalling pathway.

**Results:**

PD induced G1 phase cell cycle arrest in the MDA-MB-231 cell line. However, synchronous treatment with PD and CDDP for 24 h, treatment with PD for 24 h followed by CDDP and treatment with CDDP for 24 h followed by PD had no influence on MDA-MB-231 cell apoptosis. We further investigated the effect of PD or CDDP withdrawal on the effects of sequential treatment and found that PD treatment for 48 h followed by withdrawal for 48 h and subsequent CDDP treatment (PD-CDDP) significantly increased apoptosis and inhibited the cell viability and colony formation of MDA-MB-231 cells, while with other regimens, PD and CDDP had an additive or antagonistic response. The preferential use of PD increased DNA damage induced by CDDP, as measured through γH2AX immunofluorescence. These findings were not observed in sh-MDA-MB-231 cells, and experiments to assess cell function in MDA-MB-468 and RB-overexpressing MDA-MB-468 cells yielded similar results, which indicated that PD enhanced the sensitivity of TNBC cells to CDDP in an RB-dependent manner. In vivo, compared with single drug treatment, combination treatment inhibited tumour growth and Ki-67 expression in MDA-MB-231 xenograft models. Western blot analysis revealed that PD enhanced sensitivity to CDDP through the CDK4/6-cyclin D1-RB-E2F pathway.

**Conclusions:**

Pre-treatment with PD synchronized the tumour cell cycle through the CDK4/6-cyclin D1-RB-E2F pathway, which increased the antitumour effect of CDDP. Thus, PD-CDDP might be an effective treatment for RB-proficient TNBC patients.

## Background

Breast cancer (BC) is the most frequently diagnosed cancer in women worldwide and continues to be one of the leading causes of cancer-related deaths [1, 2]. The standard treatment for BC mainly depends on the molecular subtype of the tumour. Triple-negative breast cancer (TNBC), which lacks oestrogen receptor (ER), progesterone receptor (PR), and human epidermal growth factor receptor 2 (HER2), accounts for approximately 15–20% of all BCs. As a highly aggressive and heterogeneous tumour, it contributes significantly to tumorigenesis and resistance to chemotherapy and is thus associated with an increased risk of disease recurrence and death [3]. Moreover, improper interventions, in terms of both timing and methods, lead to earlier relapse and a worse outcome [4]. Hence, there is a desire to explore more effective strategies.

Over the last decade, advances in molecular translational research have heralded major breakthroughs in the understanding, diagnosis and management of breast cancer. Cell cycle progression undertakes a crucial role in cell proliferation, the aberration of which has been acknowledged as a hallmark of cancer [5, 6]. CDK4 and CDK6, cyclin-dependent kinases that function in the form of cyclin D1-bound compounds at a cell cycle checkpoint, can promote G1-S phase transition in the cell cycle. A major target of CDK4 and CDK6 during cell cycle progression is the tumour suppressor retinoblastoma (RB) protein. When RB is phosphorylated, its growth-suppressive properties are inactivated, releasing E2Fs. Amplification and overactivation of the CDK4/6-cyclin D1-RB-E2F pathway have been observed in various malignancies, including BC [7–12]. Selective CDK4/6 inhibitors “turn off” these kinases and dephosphorylate RB, resulting in the blockade of cell cycle progression in mid-G1 phase and preventing the proliferation of cancer cells [13].

Although many pre-clinical and clinical trials based on different TNBC subtypes have been conducted, no explicit targets have yet been identified [14, 15]. Clinical studies have confirmed the efficacy of cisplatin (CDDP), given through either single or combined administration, for TNBC. However, CDDP is highly toxic to the blood and nervous system and has limited survival benefits, so it is not routinely applied in chemotherapy regimens [16, 17]. Palbociclib (PD0332991, PD), a selective CDK4/6 inhibitor, has been approved by the Food and Drug Administration (FDA) as a first-line endocrine-based therapy for postmenopausal women with hormone-receptor-positive (HR +), HER2-negative advanced or metastatic breast cancer [14, 18–20]. Therefore, we wondered whether PD alone or in combination with CDDP could be applied as a novel treatment for TNBC. To answer this question we performed the current study to discover the effect of PD alone or combined with CDDP on TNBC cells and the corresponding mechanism.

## Methods

### Cell culture and treatments

The TNBC cell line MDA-MB-231 was kindly donated by Professor Erwei Song, University of Sun Yat-Sen in 11/2018. The TNBC cell lines MDA-MB-468 and HCC1937 were purchased from GeneChem Company (Shanghai, China) in 01/2019. All cells were free of mycoplasma contamination, and their identify was authenticated by short tandem repeat (STR) DNA profiling by Shanghai Biowing Applied Biotechnology Co., Ltd on 31/10/2019. All cells used in the experiments were within 30 passages after thawing. MDA-MB-231 cells were cultured in DMEM with 10% FBS (Gibco, Grand Island, NY, USA). MDA-MB-468 cells were cultured in RPMI-1640 medium with 10% FBS (Gibco). Cells were incubated at 37 °C in a humidified atmosphere containing 5% CO_2_. In vitro, cells were treated with 500 nM PD (PD-0332991, SelleckChem, Houston, TX, USA) or vehicle (PBS, BOSTER, Wuhan, China). MDA-MB-231 cells were treated with 50 μM CDDP, and MDA-MB-468 cells were treated with 1 μM CDDP (SelleckChem).

### Apoptosis analysis

Cells (5 × 10^4^/well) were seeded in triplicate in 10% RPMI-1640 medium/DMEM-FBS (complete medium) in 6-well plates and treated with PD and CDDP at the indicated concentrations separately or combined. After being treated for the defined duration, the cells were washed, resuspended in binding buffer, and stained with Annexin V-FITC/PI according to the manufacturer’s instructions (BD Biosciences). The apoptotic cell populations were analysed using flow cytometry (Beckman Coulter, CA). All assays were independently performed three times.

### Cell cycle analysis

For cell cycle analysis, cells were harvested, washed with PBS, fixed in pre-chilled 70% ethanol, and kept overnight at − 20 °C. The fixed cells were then collected, washed, and resuspended in PBS. The cells were incubated with 1 mg/mL RNase and 50 µg/mL propidium iodide (PI) in the dark for 30 min at 37 °C and subjected to flow cytometry (Beckman Coulter, CA). The cell cycle results were analysed using FlowJo version 7.6.1. All assays were independently performed in triplicate.

### Assessment of cell viability

The viability of the cells was assessed with Cell Counting Kit-8 reagent (CCK8, Dojindo, Tokyo, Japan). A total of 5000 to 10,000 cells per well, depending on the growth characteristics of each cell line were seeded in 96-well plates in triplicate. After adhering overnight, the designated drugs were added at different concentrations and/or sequences to the wells. After the defined duration, the supernatants were removed, and 100 μl of CCK8 solution (1:10 dilution) was added to the cells. After 2 h of incubation at 37 °C in the dark, the optical density (OD) at 450 nm was measured with a microplate reader (Bio-Rad Laboratories, Hercules, CA, USA). Each experiment was performed three times. The half maximal inhibitory concentration (IC50) was determined from dose–response curves generated by GraphPad Prism version 6.0. The combination index (CI) values for the combined treatment regimens with PD and CDDP were calculated with CompuSyn version 1.0 [21].

### Colony formation assay

Cells (500–1000/well) were seeded in 6-well plates and treated with the indicated drugs. After combination treatments, the medium was replenished every 3 days until cells in the control wells reached 80–100% confluence. The cell monolayers were then fixed and stained with a solution of 4% paraformaldehyde and 0.5% crystal violet for 30 min at room temperature, washed with water, and dried. Colonies containing 50 or more cells were visually identified and counted. Assays were performed with three independently treated cell populations.

### Tumour xenograft studies

This study was approved by the Ethics Committees of Tongji Hospital and performed in accordance with the Guide for the Care and Treatment of Laboratory Animals of Tongji Hospital. Four-week-old female BALB/c nude mice were purchased from Beijing Vital River Laboratory Animal Technology Co., Ltd., and quarantined alone for one week before the experiment. For MDA-MB-231 cell line xenograft models, 7 × 10^6^ cells were suspended in 100 μl of PBS plus 50 μl of Matrigel (BD Biosciences, MA, USA) and subcutaneously injected into the left axilla. One week later, mice bearing engrafted tumours of 50 mm^3^ were randomized to receive oral treatment with 150 mg/kg PD (n = 4), the intraperitoneal injection of 5 mg/kg CDDP (n = 4), PD-CDDP treatment (n = 4) or vehicle (PBS) treatment (n = 4) according to the dosing schedule provided in Fig. 4a. The perpendicular tumour diameters were measured with callipers. Tumour volumes were calculated as (length × width^2^)/2 every 3 days. Tumours were weighed when the mice were euthanized by cervical dislocation. All mice were sacrificed when the tumour burden of the vehicle group was equal to 1000 mm^3^. Tumours were fixed in 4% paraformaldehyde for paraffin embedding and used for immunohistochemical staining.

### Immunohistochemistry

Tissue sections were incubated with antibody against Ki-67 (#9027, 1:400) (Cell Signaling Technology) overnight at 4 °C and stained with 3,3′-diaminobenzidine (DAB). Densitometry analysis was performed using ImageJ version 1.48v.

### Quantitative real-time PCR (qRT-PCR)

Total RNA was extracted from cells using TRIzol reagent (Invitrogen, California, USA). RB expression was measured in triplicate using SYBR Green qPCR Mix (Toyobo, Shanghai, China) according to the manufacturer’s instructions. Primer sequences were as follows: RB Forward: 5′-CTCTCGTCAGGCTTGAGTTTG-3′, RB Reverse: 5′-GACATCTCATCTAGGTCAACTGC-3′; GAPDH Forward: 5′-GGAGCGAGATCCCTCCAAAAT-3′, and GAPDH Reverse: 5′-GGCTGTTGTCATACTTCTCATGG-3′. The comparative Ct method was used to calculate the relative mRNA expression, and GAPDH was used as an internal control.

### Cell transfection

Plasmids containing small hairpin RNA (shRNA) targeting RB and negative control shRNA (shNC), RB-overexpressing plasmid and empty plasmid were purchased from RiboBio (Guangzhou, China). ShRNA was transfected into MDA-MB-231 cells, and RB-overexpressing plasmid and empty plasmid were transfected into MDA-MB-468 cells with X-tremeGENE HP DNA transfection reagent (Roche, CHE) according to the manufacturer’s instructions. The shRNA sequences were as follows: shNC, 5′-TTCTCCGAACGTGTCACGT-3′, shRB, 5′-CGGCTAAATACACTTTGTGAA -3’.

### Immunofluorescence assay

Breast cancer cells were subjected to indirect immunofluorescence staining with γH2AX (Ser139, #9718, 1:400) and then labelled with FITC goat anti-rabbit IgG (#AS007, 1:200). Nuclei were stained with DAPI (Life Technology). Fluorescence images were acquired using an inverted fluorescence microscope (Olympus). ImageJ version 1.48v was utilized for foci measurement and image analysis.

### Western blot analysis

Cell lysates were separated by SDS-PAGE, and the proteins were then transferred to PVDF membranes. The proteins were detected using antibodies against the following: RB (ab181616, 1:2000), cyclin D1 (ab40754, 1:1000), E2F1 (ab179445, 1:1000) (Abcam, Cambridge, UK), phospho-RB (p-RB) (S780) (#8180, 1:1000), PARP/cleaved PARP (#9542, 1:1000) (Cell Signaling Technology), and β-Actin (AC026, 1:100,000) (ABclonal, Boston, USA). Specific bands were visualized by ECL (Advansta, USA) and detected with an imaging system (Bio-Rad, USA).

### Statistical analysis

Statistical significance was determined and means and standard deviations were calculated by GraphPad Prism version 6.0. All analyses were performed in triplicate, and *P* < 0.05 was used to indicate statistical significance. Data are expressed as the mean ± SD or mean. The significance of differences between two groups was analysed by two-tailed Student’s *t*-test. The Chou-Talalay method was performed to calculate the CI.

## Results

### Mutations and dysregulation of genes in the CDK4/6 pathway are common in TNBC

**Fig. 1:**
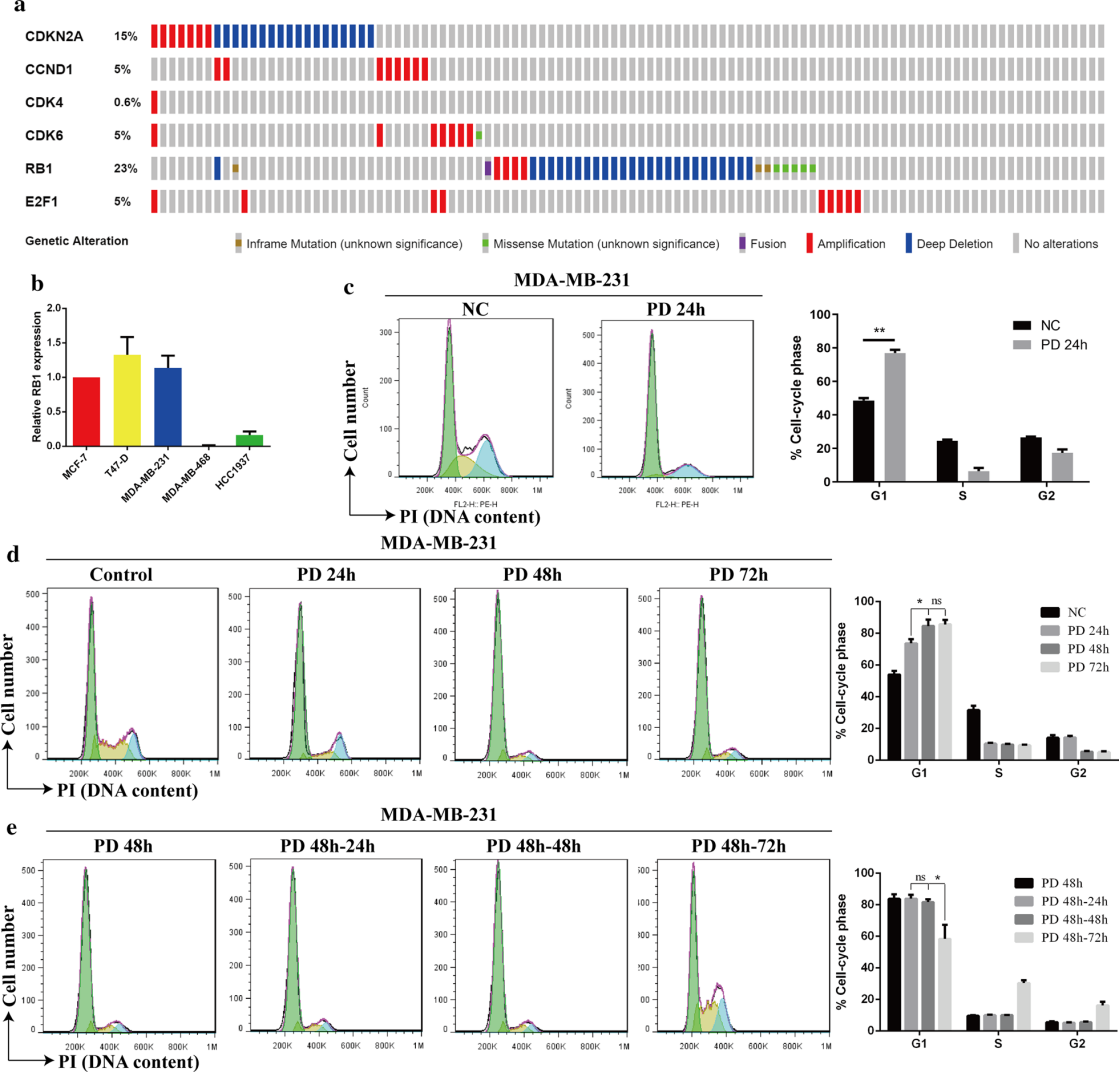
PD is a potential therapeutic treatment for TNBC. **a** Analysis of 171 tumours from the TCGA database showing mutations of genes in the CDK4/6 pathway. **b** The mRNA expression of RB in different breast cancer cell lines. **c** Cell cycle analysis of MDA-MB-231 cells treated with PD for 24 h. **d** MDA-MB-231 cells were treated with PD for 24 h, 48 h, or 72 h. **e** MDA-MB-231 cells were treated with PD for 48 h, after which PD was withdrawn for 24 h, 48 h, or 72 h. Changes in the cell cycle were observed by flow cytometry. Student’s *t*-test (two-tailed, equal variance) was used to derive *P* values: ^*^P < 0.05, ^**^P < 0.01, ^***^P < 0.001

TNBC genomic mutations in the CDK4/6 pathway from The Cancer Genome Atlas (TCGA) were obtained and visualized by the cBioPortal browser (www.cbioportal.org) [22, 23]. CDKN2A, also known as multiple tumor suppressor l (MTS1), acts to stop cell cycle progression by inhibiting CDK4 and CDK6. As shown in Fig. 1a, 15% of TNBC patients showed CDKN2A amplification or deep deletion. Twenty-three percent of patients showed significant amplification, deep deletion or other mutations in the RB gene. Other genes in the CDK4/6 pathway were mainly amplified in TNBC patients. All of these mutations would contribute to disorder of the CDK4/6 pathways and uncontrolled cell cycle. In general, these data indicated that therapy with a CDK4/6 inhibitor would likely benefit some TNBC patients. Therefore, we investigated the effects of the CDK4/6 inhibitor PD on TNBC cells. Since previous studies have shown that the RB gene plays an important role in the mechanism of CDK4/6 inhibitors [18–20], we detected the mRNA levels of RB in different breast cancer cell lines. As a result, the mRNA level of RB was significantly higher in MDA-MB-231 cells than in the other TNBC cell lines, and RB mRNA levels in this cell line were basically as high as those in HR + cell lines (MCF-7 and T47-D) (Fig. 1b). Therefore, we chose MDA-MB-231 cells for the following experiments.

### PD led to G1 phase arrest in MDA-MB-231 cells and three drug regimens were initially established

As expected, in MDA-MB-231 cells, PD significantly blocked the cell cycle in G1 phase (Fig. 1c). Then, we found that PD had no significant effect on the apoptosis of MDA-MB-231 cells after its continuous application for 24 h, 48 h or 72 h (Additional file 1: Figure S1A). Therefore, we next investigated the possibility that PD enhances the sensitivity of TNBC cells to CDDP. We established three common drug regimens based on literatures: PD and CDDP (synchronous treatment with PD and CDDP for 24 h), PD to CDDP (PD for 24 h followed by CDDP for 24 h), and CDDP to PD (CDDP for 24 h followed by PD for 24 h) (Additional file 2: Figure S2A–C). However, none of these regimens significantly increased MDA-MB-231 cell apoptosis compared with that in the group treated with CDDP alone (Additional file 1: Figure S1B).

### Establishment of three novel drug regimens according to the effect of PD on the cell cycle

To discover an effective drug regimen in MDA-MB-231 cells, we further investigated the effects of PD on the cell cycle. With prolonged PD treatment, its effect in blocking the cell cycle in MDA-MB-231 cells was gradually strengthened, which was manifested as a gradual increase in the proportion of cells at G1 phase and gradual decreases in the proportions of cells at G2 and S phases. When PD treatment continued for 48 h, the proportion of cells at G1 phase peaked, as it was not significantly increased when the treatment duration was extended to 72 h (Fig. 1d). We continued to investigate the effects of PD withdrawal on the cell cycle after 48 h of continuous treatment with PD and found that when MDA-MB-231 cells were continuously treated with PD for 48 h and the PD was then withdrawn for 48 h, the ratio of cells in G1 phase began to decrease (Fig. 1e), indicating that the inhibited cells had re-entered the cell cycle. According to the above data, we set three novel drug regimens: PD + CDDP, CDDP-PD and PD-CDDP. PD + CDDP treatment was performed by treating cells with PD for 48 h with concomitant CDDP treatment for the first 24 h. CDDP-PD treatment was performed by treating cells with CDDP for 24 h and then withdrawing CDDP for 48 h before PD exposure for 48 h. PD-CDDP treatment was performed by treating cells with PD for 48 h and then withdrawing PD for 48 h before CDDP exposure for 24 h (Additional file 2: Figure S2D–F).

### PD-CDDP treatment had a synergistic inhibitory effect on cell functions in MDA-MB-231 cells in vitro

**Fig. 2:**
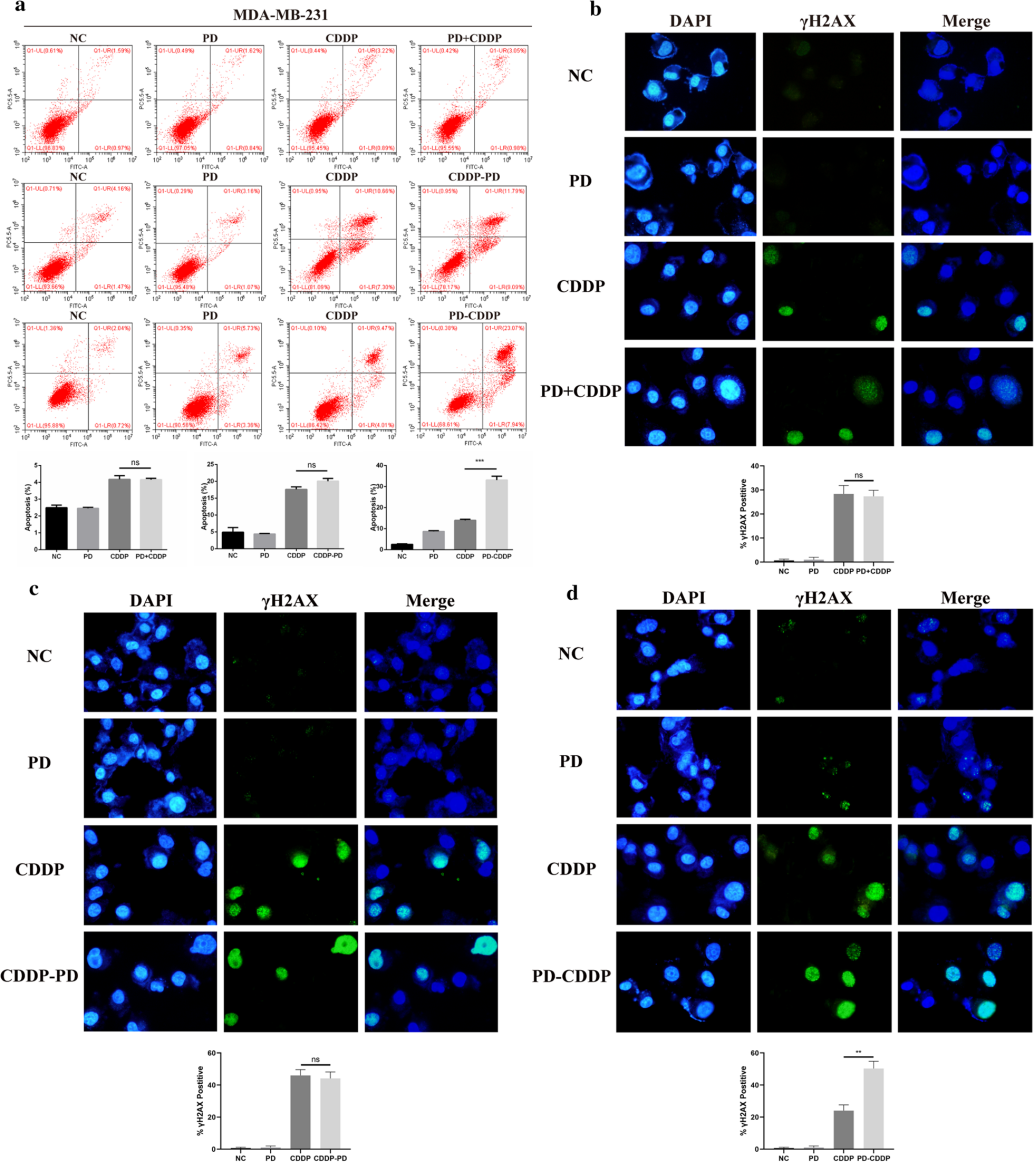
PD enhanced CDDP-induced apoptosis and DNA damage in MDA-MB-231 cells in the PD-CDDP group. **a** Flow cytometry showed that PD enhanced the CDDP-induced apoptosis of MDA-MB-231 cells in the PD-CDDP group, and the average percentage of cells in apoptosis was quantified. **b**–**d** Immunofluorescence analysis was used to evaluate the expression of γH2AX (green) in MDA-MB-231 cells treated with PD, CDDP, PD + CDDP, PD-CDDP and CDDP-PD. DAPI was used to detect nuclei. Images were captured at × 400 with an Olympus microscope

**Fig. 3:**
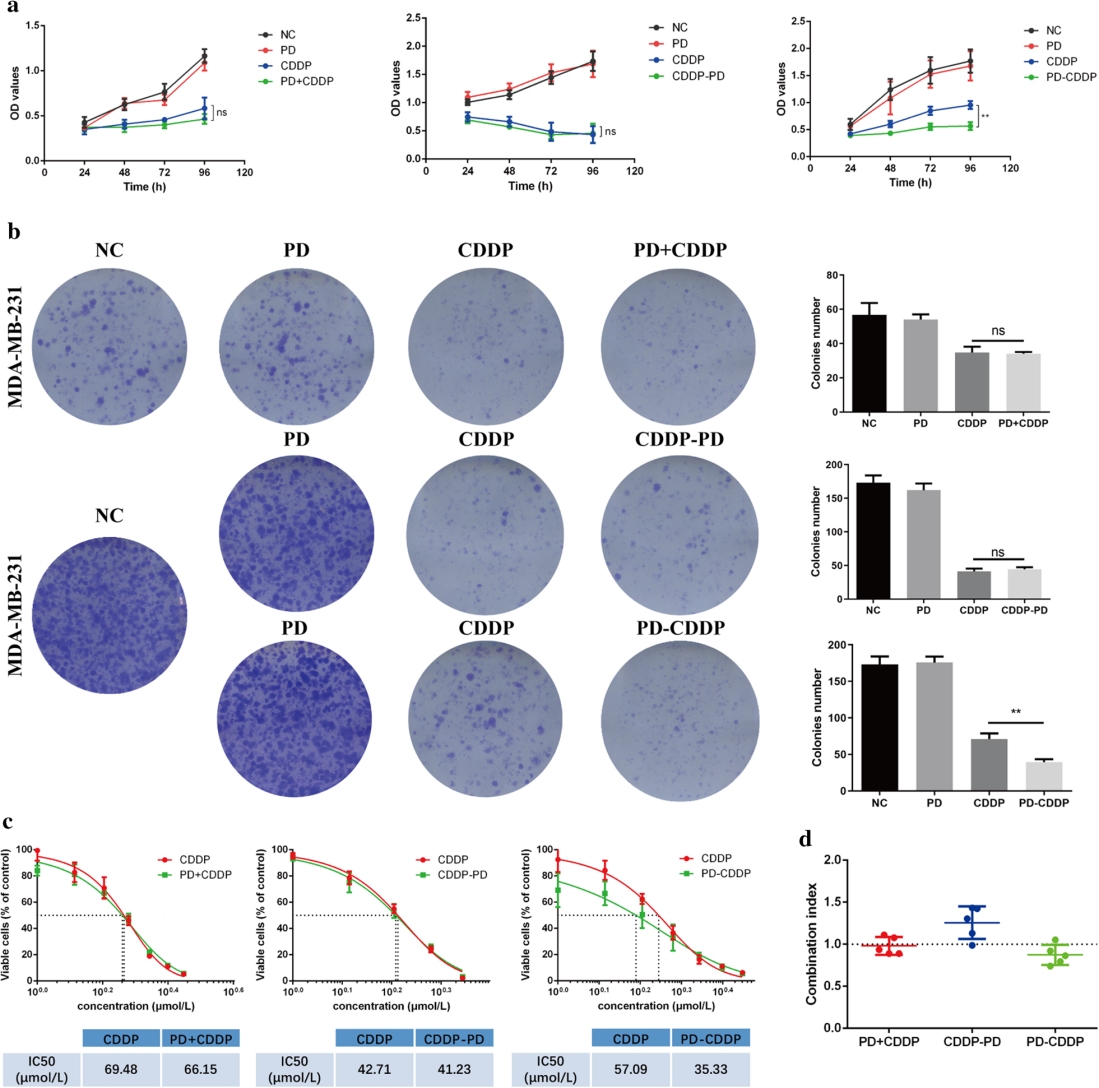
PD enhanced the sensitivity of MDA-MB-231 cells to CDDP. **a** The cell viability of MDA-MB-231 cells after exposure to each treatment regimen was determined by CCK8 assay. **b** A colony formation assay and its quantification of MDA-MB-231 cells after each treatment regimen had been performed. **c** The IC50 of CDDP (drug concentration required to inhibit growth by 50%) was calculated in MDA-MB-231 cells treated with PD + CDDP, PD-CDDP or CDDP-PD. **d** Combination index (CI) obtained from dose–response analysis of the CDDP and PD treatments. The data, which were analysed with CompuSyn software, were used to measure whether the two drugs had a synergistic (CI < 1), additive (CI = 1) or antagonistic (CI > 1) effect

There was no significant difference in the apoptosis of MDA-MB-231 cells between the PD + CDDP group and CDDP alone group, or between the CDDP-PD group and CDDP alone group. However, the apoptosis of MDA-MB-231 cells treated with PD-CDDP was significantly increased compared with that of cells treated with CDDP alone (Fig. 2a). Previous studies have demonstrated γH2AX foci to be an accurate readout for double-strand DNA breaks. PD + CDDP and CDDP-PD treatment had no obvious effect on DNA damage compared with the results of CDDP treatment alone (Fig. 2b, c), while MDA-MB-231 cells treated with PD-CDDP exhibited increased γH2AX positivity (Fig. 2d). Moreover, the PD-CDDP group had lower cell viability and formed fewer colonies than the CDDP alone group (Fig. 3a, b). Measurement of IC50 of CDDP showed that when sequentially administered before CDDP, PD sensitized MDA-MB-231 cells to CDDP (Fig. 3c), which indicated that PD-CDDP treatment could reduce the dose of CDDP. CompuSyn software was used to calculate the CI values. CI values of 1 and > 1 are indicative of additive and antagonistic responses, respectively, whereas CI values < 1 indicate a synergistic response. The CI values further indicated that the optimal order for combination treatment with the two drugs was PD-CDDP, which maximized the inhibitory effect on MDA-MB-231 cells (Fig. 3d).

### PD-CDDP treatment effectively delayed the proliferation of MDA-MB-231 xenografts in vivo

**Fig. 4:**
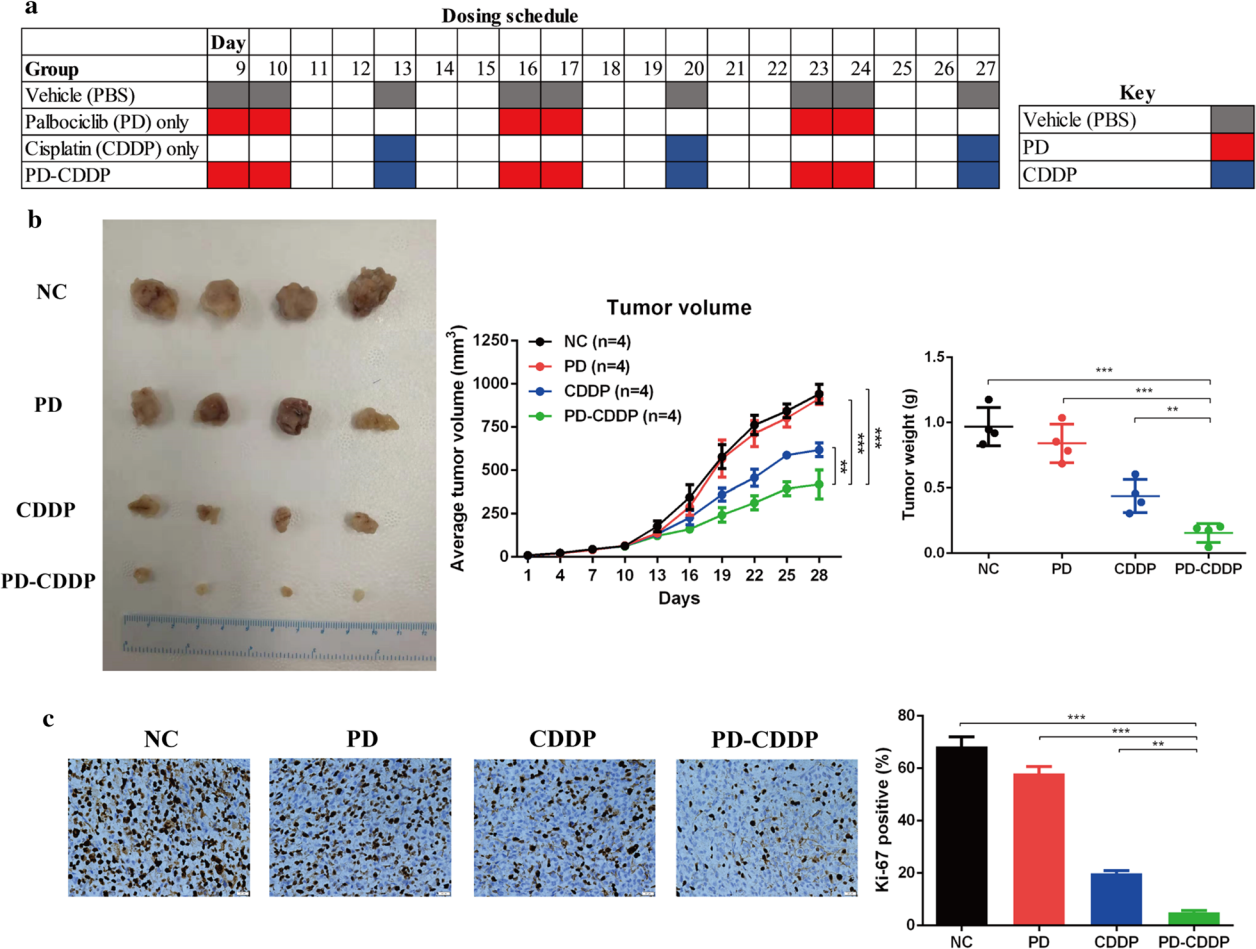
PD-CDDP treatment effectively delayed the proliferation of MDA-MB-231 xenografts in vivo. **a** Sample dosing schedule for PD and CDDP. **b** Randomly grouped nude mice bearing MDA-MB-231 tumours were treated with vehicle (PBS), PD (150 mg/kg), CDDP (5 mg/kg), or PD-CDDP treatment for three cycles. Tumour volume was determined every 3 days after the onset of treatment. **c** Harvested tumours were subsequently collected, and representative immunohistochemistry results show Ki-67 expression in MDA-MB-231 tumours. The percentage of Ki-67-positive tumour cells and their staining intensities were assessed by ImageJ

Sixteen female BALB/c nude mice at five weeks of age were given 7 × 10^6^ suspended MDA-MB-231 cells. After 1 week, they were randomly divided into four groups, and the treatment schemes are shown in Fig. 4a. As a result of treatment, the tumour volumes and weights in the PD-CDDP group were significantly lower than those in the control group (Fig. 4b). Immunohistochemical analysis showed that the expression of Ki-67 in the PD-CDDP group was significantly lower than that in the control group (Fig. 4c). Collectively, these results indicate that the application of PD before CDDP significantly delayed tumour growth, which was consistent with the results of in vitro experiments.

### PD synergistically enhanced CDDP-mediated cytotoxicity in an RB-dependent manner

**Fig. 5:**
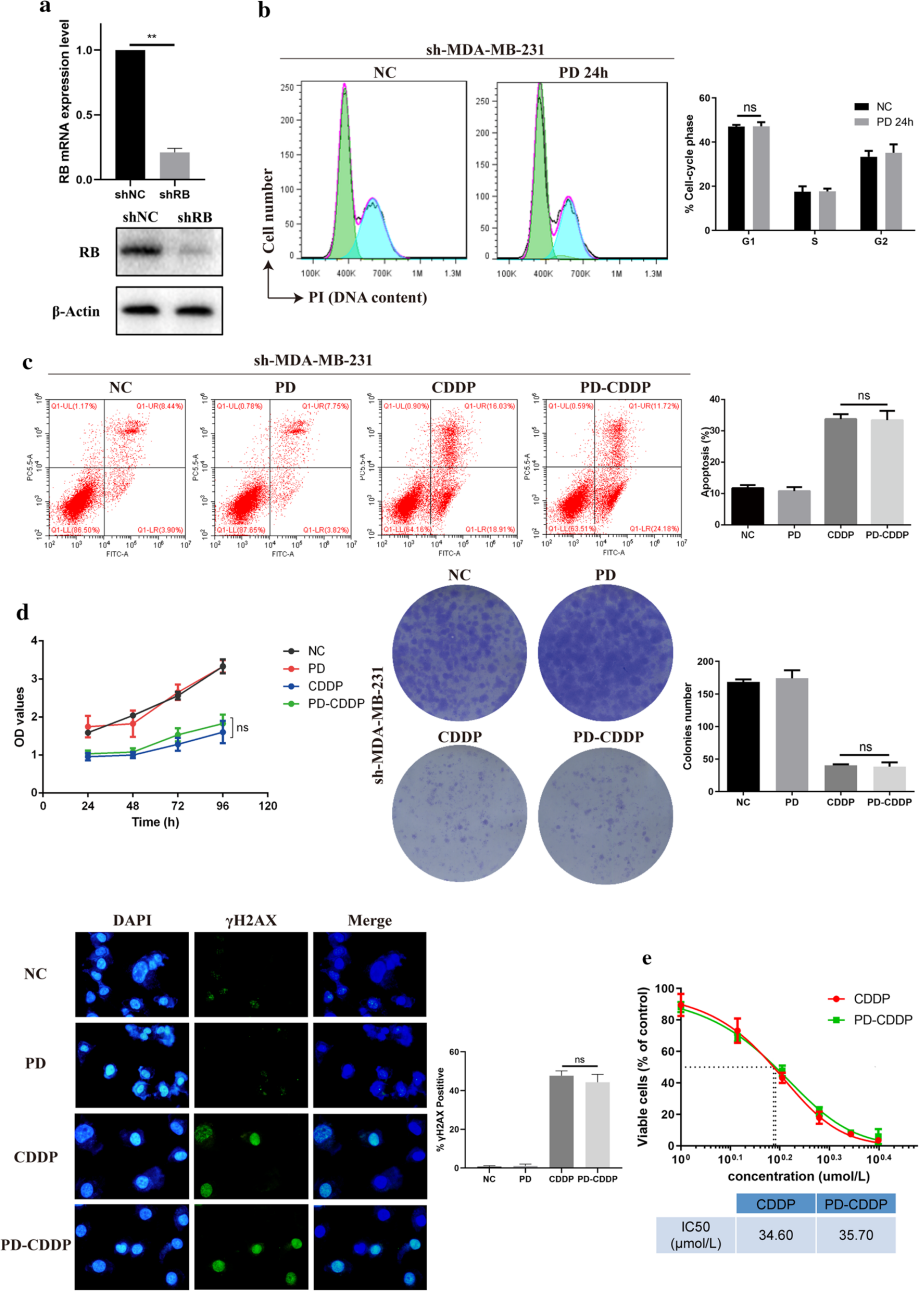
PD synergistically enhanced CDDP-mediated cytotoxicity in a potentially RB-dependent manner. **a** RB mRNA and protein expression levels in MDA-MB-231 cells were dramatically decreased by shRB, according to the results of RT-PCR and Western blot analysis. **b** Cell cycle analysis of sh-MDA-MB-231 cells treated with PD for 24 h. **c** Flow cytometry showed that PD did not enhance the CDDP-induced apoptosis of sh-MDA-MB-231 cells, and the average percentage of cells in apoptosis was quantified. **d** CCK8 assay, colony formation assay and immunofluorescence analysis were performed and showed that PD did not significantly enhance the CDDP-induced inhibition of cell viability or colony formation or increase in DNA damage in sh-MDA-MB-231 cells. **e** The IC50 of CDDP was calculated in sh-MDA-MB-231 cells treated with PD-CDDP

**Fig. 6:**
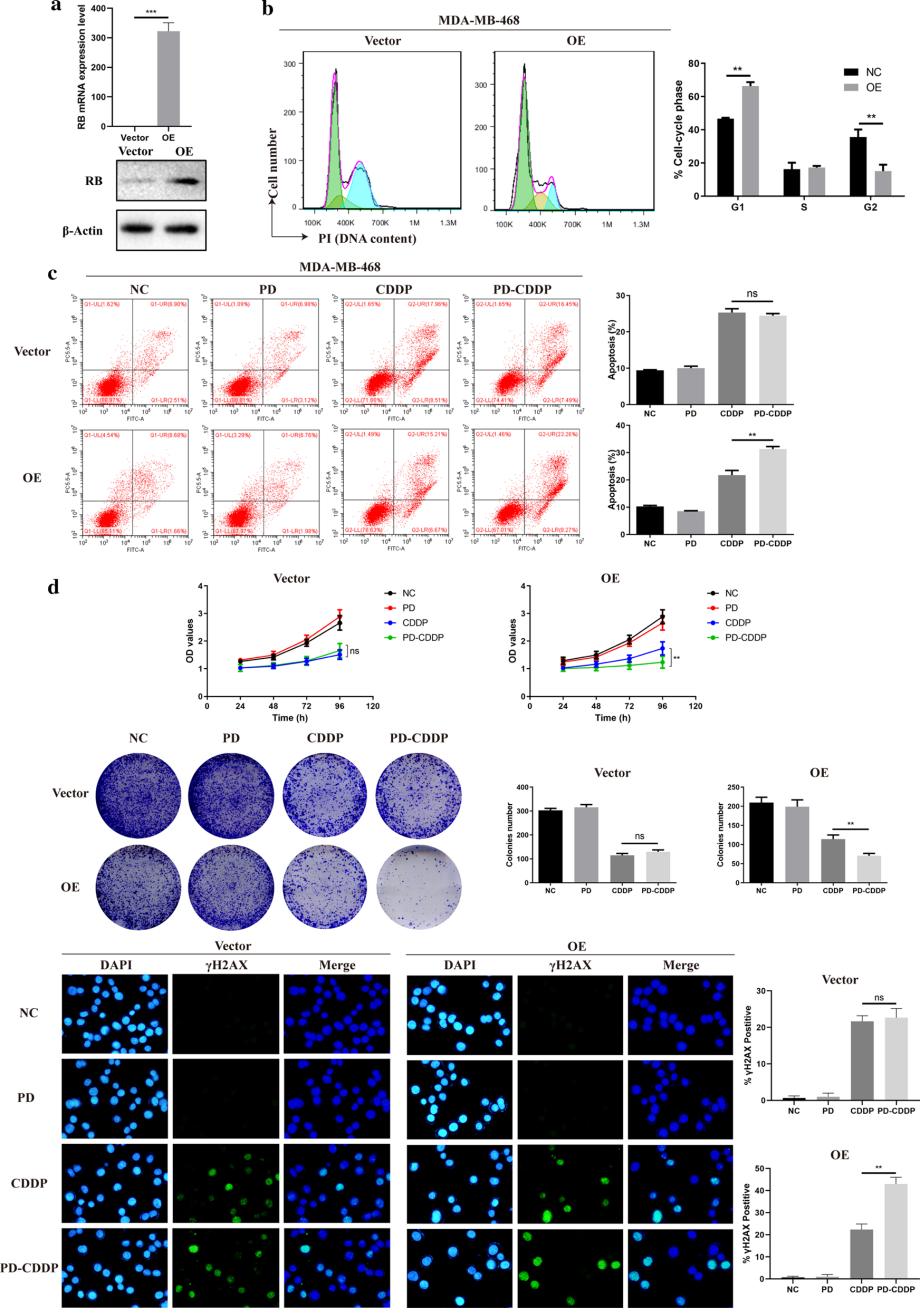
Further validation of the RB-dependent synergistic effect of PD on CDDP- induced cytotoxicity in MDA-MB-468 cells. **a** RB mRNA and protein expression levels were significantly higher in RB-overexpressing MDA-MB-468 (OE) cells than in MDA-MB-468 cells transfected with empty plasmid (vector), according to results of RT-PCR and Western blot analysis. **b** Cell cycle analysis of OE and vector cells treated with PD for 24 h. **c** Flow cytometry showed that PD enhanced the CDDP-induced apoptosis of OE cells but not vector cells in the PD-CDDP group, and the average percentage of cells in apoptosis was quantified. **d** A CCK8 assay, colony formation assay and immunofluorescence analysis were performed to determine the cell viability, colony formation and DNA damage, respectively, in OE and vector cells

The mRNA and protein expression levels of RB in MDA-MB-231 cells were dramatically decreased by shRB (Fig. 5a). The treatment of MDA-MB-231 cells with PD resulted in prominent G1 cell cycle arrest, as described above, but no such changes were found in RB-knockdown MDA-MB-231 (sh-MDA-MB-231) cells (Fig. 5b). The apoptosis of sh-MDA-MB-231 cells treated with PD-CDDP showed no significant difference compared with that of cells treated with CDDP alone (Fig. 5c). Likewise, among sh-MDA-MB-231 cells, there were no obvious differences between the PD-CDDP group and corresponding CDDP alone group in terms of cell viability, colony formation or DNA damage (Fig. 5d). Measurement of the IC50 of CDDP showed that PD could not change the sensitivity of sh-MDA-MB-231 cells to CDDP (Fig. 5e). Since the relative mRNA expression of RB in MDA-MB-468 cells was lowest, we constructed RB-overexpressing MDA-MB-468 (OE) cells for further verification. The vector cells refers to MDA-MB-468 cells transfected with empty plasmid. As shown in Fig. 6a, RB expression at both the mRNA and protein levels was significantly higher in OE cells than in vector. The treatment of OE cells with PD resulted in obvious G1 cell cycle arrest, as seen in MDA-MB-231 cells (Fig. 6b). Among OE cells, apoptosis was significantly higher in the PD-CDDP group than in the CDDP alone group, but no such difference was observed in vector cells (Fig. 6c). Similarly, PD-CDDP treatment significantly inhibited cell viability and colony formation and increased DNA damage in OE cells compared with the corresponding group treated with CDDP alone, but these differences were not observed in vector cells (Fig. 6d). Therefore, we speculated that PD-CDDP treatment had inhibitory effects on RB-proficient TNBC cells but no such effects on RB-deficient TNBC cells.

### PD-CDDP treatment enhanced the inhibitory effect of CDDP through the cyclin D1/RB/E2F axis

**Fig. 7:**
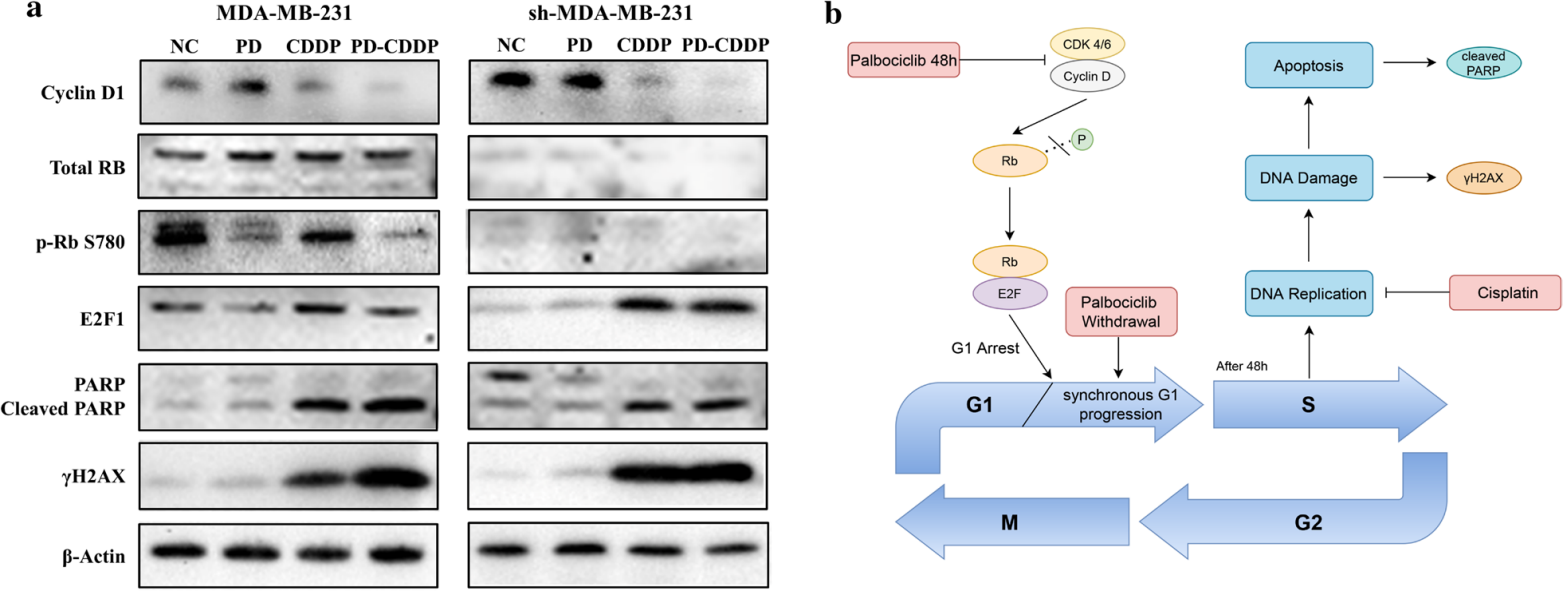
**a** PD-CDDP treatment enhanced the antitumour effect of CDDP through the cyclin D1/RB/E2F axis. Western blot analysis was performed to assess cyclin D1, total RB, p-RB, E2F1, PARP/cleaved PARP, γH2AX and β-actin expression. **b** Proposed model for the mechanism of PD-CDDP treatment

Consistent with the above results, the total RB protein level was obviously higher in MDA-MB-231 cells. Furthermore, Western blot analysis revealed that compared with MDA-MB-231 cells in the CDDP alone group, those in the PD-CDDP group exhibited significantly reduced levels of p-RB, and the downstream transcription factor E2F1 was also significantly reduced, but no such change was observed in sh-MDA-MB-231 cells (Fig. 7a). This finding indicated that PD inhibited the cyclin D1/RB/E2F pathway in RB-proficient cells and increased CDDP-induced apoptosis and DNA damage of MDA-MB-231 cells, which resulted in increased cleaved PARP and γH2AX levels. A proposed mechanistic model is shown in Fig. 7b.

## Discussion

In this study, we explored the effect of various combinations of PD and CDDP in the treatment of TNBC. We initially established three common drug regimens and discovered that compared with CDDP treatment alone, the simply combined or sequential use of PD and CDDP was no more effective in MDA-MB-231 cells. Therefore, we performed a deeper investigation of the drug regimens and found synergism in the PD-CDDP group. Based on the effect of PD on the cell cycle, when PD was used for 48 h and then withdrawn for 72 h, its effect in blocking the cell cycle was weakened, and some cells entered S and G2 phase. Therefore, it is reasonable to speculate that when PD is used for 48 h and then withdrawn for 48 h, most cells are synchronized in the cell cycle and ready to enter S phase, in which cells are more sensitive to CDDP, thereby achieving the synergistic effect observed in the PD-CDDP group. In contrast, tumour cells in the CDDP-PD group were at various stages of the cell cycle, which show quite different sensitivities to CDDP. Sequential use of PD following CDDP may induce the recovery of partial cells with DNA damage due to cell cycle extension, so CDDP-PD treatment ultimately produced an effect antagonistic to that of CDDP alone or the same effect as CDDP alone.

Combination therapy that relies on complementary mechanisms of antitumour activity has increasingly become a trend in cancer treatment [24, 25]. Currently, an increasing number of targeted therapies, such as CDK inhibitors, combined with conventional chemotherapy regimens have been applied to improve the antitumour effects of the individual therapies and inhibit tumour resistance. For example, pre-treatment with PD could sensitize myeloma cells to bortezomib-induced apoptosis [26]. Another study found that simultaneous combination treatment consisting of abemaciclib with paclitaxel or CDDP could achieve better efficacy than chemotherapy alone [27]. In contrast with the above findings, Patrick et al. demonstrated that simultaneous combination treatment of carboplatin with PD decreased antitumour activity compared with carboplatin treatment alone in Rb-proficient mice and that the coadministration of PD with carboplatin had no effect on tumour growth in vivo [28]. The above results indicate that care is required when combination treatments consisting of CDK inhibitors and chemotherapeutic drugs are designed. Different tumour types, the use of different chemotherapeutic agents, and even the use of different time points, as shown in our study, may have totally different effects. Improper strategies may be ineffective or even have results opposite of the intended results.

In addition, we demonstrate that the synergistic effect of PD with CDDP-mediated cytotoxicity occurs in an RB-dependent manner. In RB-knockdown or RB-deficient cell lines, PD could not induce cell cycle arrest, and PD-CDDP could not enhance the cytotoxic effect of CDDP, but the overexpression of RB restored the sensitivity of RB-deficient cells to PD. Some studies and our study have demonstrated that RB can act as a marker to select patients suffering from cancer who are likely to benefit from PD treatment, and the loss of RB function may be the main cause of primary and secondary drug resistance to PD [29–31].

Overall, this is the first study to investigate the inhibition of TNBC cells by a combination strategy consisting of PD and CDDP under a specific drug regimen. However, there are several limitations in this study. We used only one RB-proficient cell line in this study, so we cannot rule out that the observed synergistic effect is specific to only MDA-MB-231 cells. Furthermore, the additional validation of CDDP-resistant cell lines in this study would have increased the clinical relevance of the study.

## Conclusions

Our data have proven that PD-CDDP treatment could significantly suppress viability and induce apoptosis and DNA damage in RB-proficient TNBC cells compared with CDDP treatment alone and that these antitumour effects can be ascribed to synchronization of the cell cycle by PD through the CDK4/6-cyclin D1-RB-E2F pathway. PD-CDDP may be a promising antineoplastic regimen in the future; however, challenges warrant further validation in prospective studies.

## Supplementary information

**Additional file 1:** **Figure S1. a** Assessment of the apoptosis of MDA-MB-231 cells treated with PD for 24 h, 48 h, or 72 h. **b** Assessment of the apoptosis of MDA-MB-231 cells treated with the three initial drug regimens.

**Additional file 2:** **Figure S2. **Different PD and CDDP drug regimens:** a** PD and CDDP,** b** PD to CDDP,** c** CDDP to PD,** d** PD+CDDP,** e** CDDP-PD and** f** PD-CDDP

## Data Availability

The datasets used and/or analyzed during the current study are available from the corresponding author on reasonable request.

## Abbreviations

PD: Palbociclib
CDDP: Cisplatin
PD-CDDP: Treating cells with PD for 48 h and then withdrawing PD for 48 h before CDDP exposure for 24 h
CDDP-PD: Treating cells with CDDP for 24 h and then withdrawing CDDP for 48 h before PD exposure for 48 h
PD + CDDP: Treating cells with PD for 48 h with concomitant CDDP treatment for the first 24 h
BC: Breast cancer
TNBC: Triple-negative breast cancer
ER: Estrogen receptor
PR: Progesterone receptor
HER2: Human epidermal growth factor receptor 2
RB: Retinoblastoma
STR: Short tandem repeat
CI: Combination index
