# Supplementary figures and images for: Novel sequential treatment with palbociclib enhances the effect of cisplatin in RB-proficient triple-negative breast cancer
Source: Cancer Cell Int. 2020 Oct 12;20:501. doi: 10.1186/s12935-020-01597-x (PMC7552520; doi:10.1186/s12935-020-01597-x)

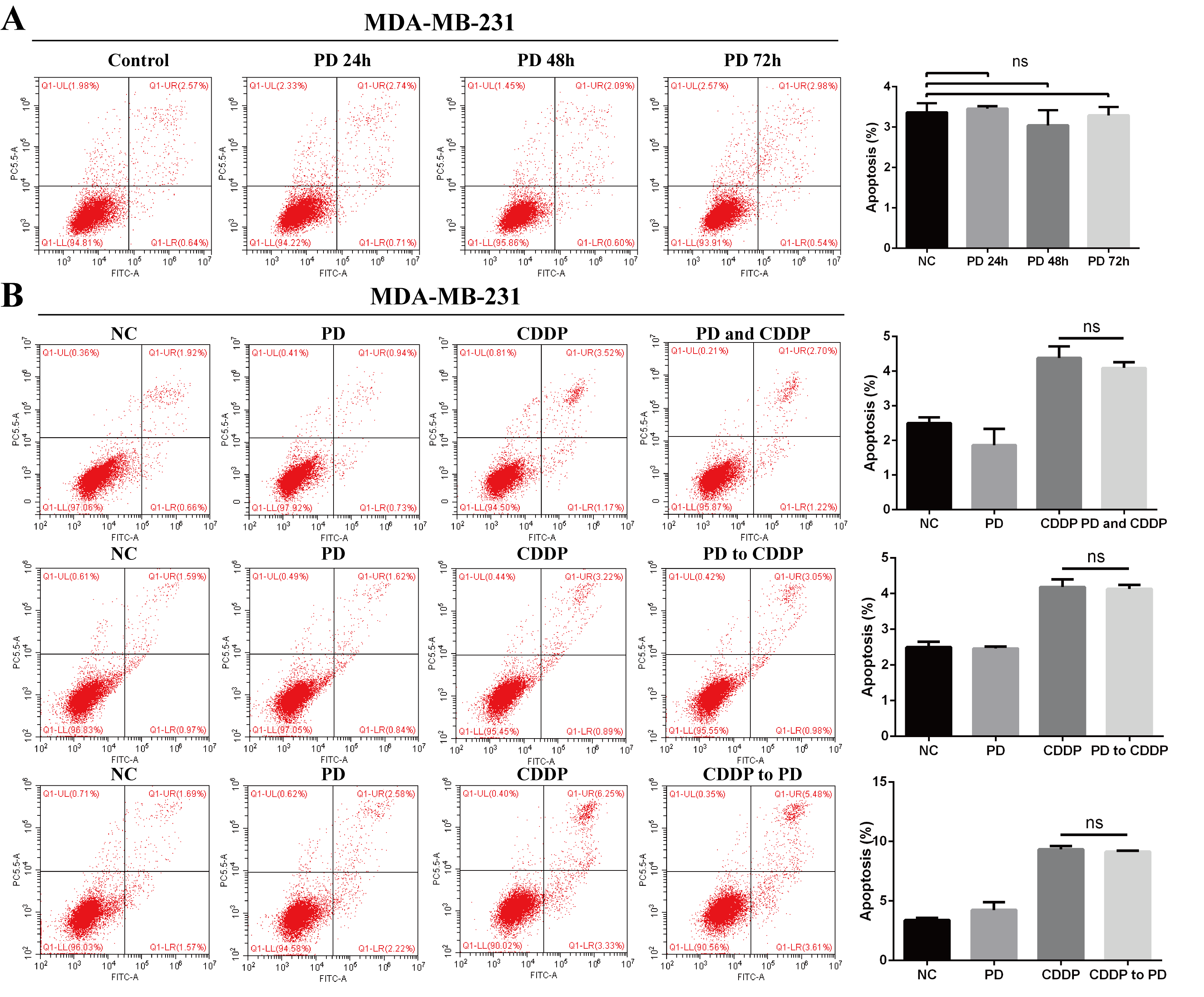

Supplement: Supplementary file 1 — Additional file 1: Figure S1. a Assessment of the apoptosis of MDA-MB-231 cells treated with PD for 24 h, 48 h, or 72 h. b Assessment of the apoptosis of MDA-MB-231 cells treated with the three initial drug regimens. [file 12935_2020_1597_MOESM1_ESM.tif]

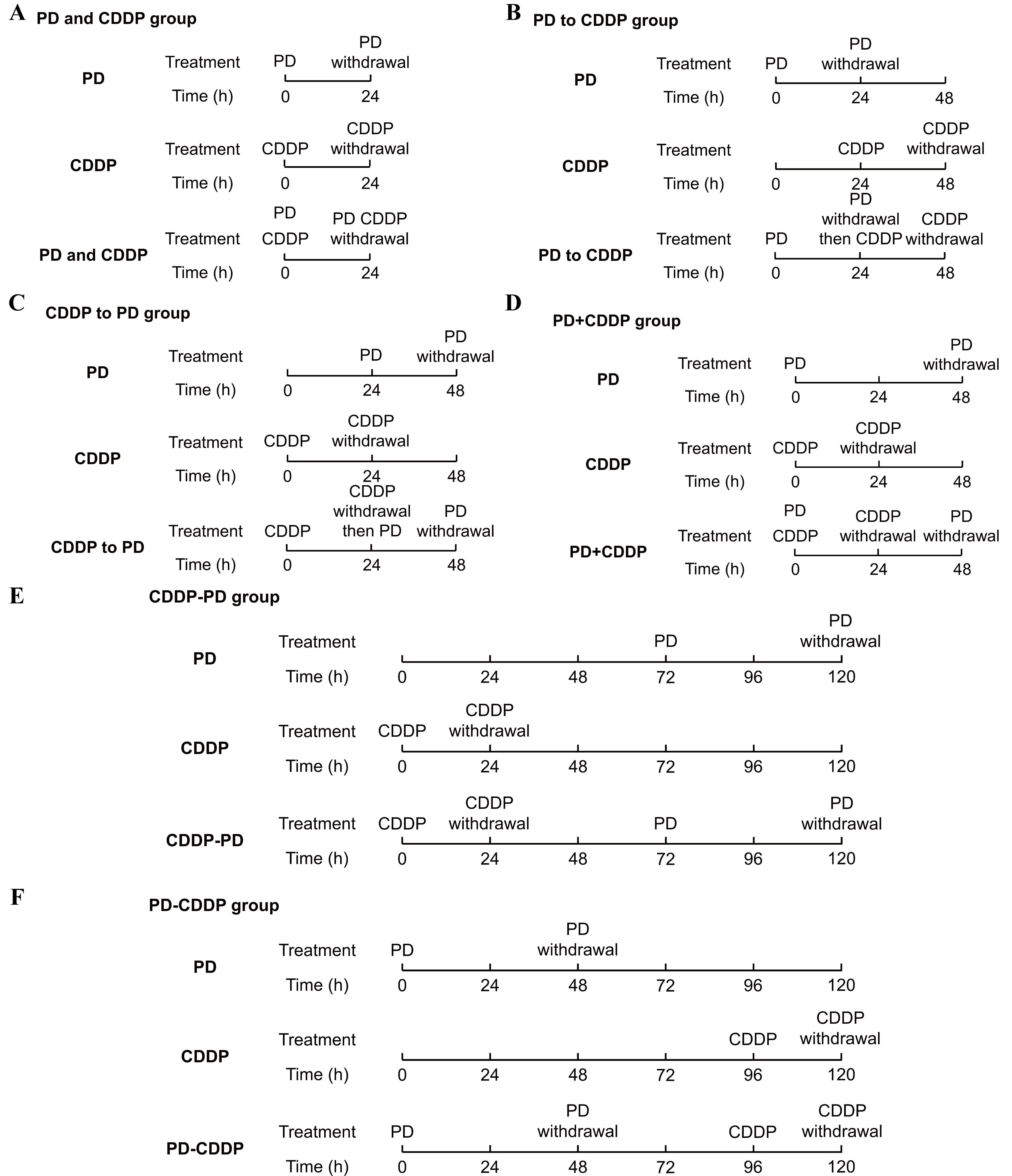

Supplement: Supplementary file 2 — Additional file 2: Figure S2. Different PD and CDDP drug regimens: a PD and CDDP, b PD to CDDP, c CDDP to PD, d PD+CDDP, e CDDP-PD and f PD-CDDP [file 12935_2020_1597_MOESM2_ESM.tif]
